# A randomized controlled trial of a six-session cognitive behavioral treatment of emotional disorders in adolescents 14–17 years old in child and adolescent mental health services (CAMHS)

**DOI:** 10.1186/s40359-020-0393-x

**Published:** 2020-03-14

**Authors:** Veronica Lorentzen, Kenneth Fagermo, Bjørn Helge Handegård, Ingunn Skre, Simon-Peter Neumer

**Affiliations:** 1 Department of Psychology, Faculty of Health Sciences, UIT The Arctic University of Norway, 9037 Tromsø, Norway; 2 Department of Child and Adolescent Psychiatry, Divisions of Child and Adolescent Health, University Hospital of North Norway, P.O. Box 19, 9038 Tromsø, Norway; 3 Regional Centre for Child and Youth Mental Health and Child Welfare, UIT The Arctic University of Norway, 9037 Tromsø, Norway; 4 Department of General Psychiatry, University Hospital of North Norway, P.O. Box 6124, 9291 Tromsø, Norway; 5 Regional Centre for Child and Adolescent Mental Health - Eastern and Southern Norway, 0484 Oslo, Norway

**Keywords:** Cognitive behavioural therapy, Adolescence, Emotional disorders, Treatment, Effectiveness, Transdiagnostic

## Abstract

**Background:**

This study aims to investigate effectiveness of a 6-week, transdiagnostic cognitive behavioral therapy (CBT) for anxiety and depression in adolescents, the Structured Material for Therapy (SMART), in naturalistic settings of child and adolescent mental health outpatient services (CAMHS).

**Methods:**

A randomized controlled trial with waiting list control (WLC) was performed at three community CAMHS in Norway. Referred adolescents (*N* = 163, age = 15.72, 90.3% girls) scoring 6 or more on the emotional disorders subscale of the Strengths and Difficulties Questionnaire (SDQ) were randomly assigned to SMART or to WLC.

**Results:**

In the treatment group (CBT), 32.9% improved in the main outcome measure (SDQ), compared to 11.6% in the WLC. Clinically significant and reliable change was experienced by 17.7% in the CBT condition, compared to 5.8% in the WLC. No patients deteriorated. Statistically significant treatment effects were achieved for internalization symptoms, anxiety symptoms and general functioning.

**Conclusions:**

These promising findings indicate that SMART may be considered as a first step in a stepped care model for anxiety and/or depression treatment in CAMHS. The recovery rates imply that further investigations into the effectiveness of brief treatments should be made. Furthermore, there is a need for more comprehensive second-stage treatments for some of these patients.

**Trial registration:**

ClinicalTrials.gov Identifier: NCT02150265. First registered May 292,014.

## Background

Anxiety and depression are the most frequently diagnosed mental health disorders, both in the general population, and consequently also in child and adolescent mental health outpatient services (CAMHS) [1–3]. In the general population up to 10% of children and 20% of adolescents will meet the criteria of an anxiety disorder at any point in time [4]. Adolescents are at high risk for the development of depression. The percentage of adolescents with major depressive disorder range from 8 to 20% before the age of 18 [5–8]. Emotional disorders interfere negatively with various aspects of functioning and quality of life [9–13]. The prevalence of both anxiety and depressive disorders increase during adolescence [14, 15]. Comorbidity and co-occurrence of anxiety and depression is high [16] and studies show that anxiety and depression both have shared and separate features and etiology [16]. Hence, combined treatments for emotional disorders could offer effective treatments for these complex disorders.

Cognitive behavioral therapy (CBT) and interpersonal treatment (IPT) are *well-established* interventions for adolescent depression [17], and numerous studies have also demonstrated that CBT relieves anxiety symptoms in youths [18, 19]. In a comprehensive multilevel meta-analysis [20], integrating the results of 140 studies from the past five decades (1963–2013), youth psychotherapies showed a significant post-treatment effect size (ES) of 0.46. For the separate disorders, the largest ES was reported for anxiety (0.61), while treatments of depression in youths have yielded weaker ES (0.29) [20]. Other systematic reviews and meta-analyses examining the effect of youth CBT, show moderate to large treatment effects on anxiety and depression in youths [21, 22]. The majority of the evidence-based protocols for youths target symptoms of single disorders [23, 24], or symptom domains [25]. So far, the most well-known and well-studied combined treatment for the comorbid features of emotional disorders is the unified protocol for treatment of emotional disorders in adolescents [26]. Although not all studies have found a relationship between treatment outcome and comorbidity [22], some have found that comorbidity predicts poorer response to interventions in youth with both primary anxiety [27–29] and primary depression [16]. According to the previously mentioned multilevel meta-analysis [20], treatments of concurrent multiple problems, as opposed to any single targeted problem, showed an effect that was not significantly different from zero at post-treatment or follow-up [20]. Some argue that this could suggest that efforts made to concurrently treat multiple problems have been less effective than focusing more narrowly [30], suggesting new ways to address comorbidity in youths [31–33]. In an earlier review of trials of 461 youth psychotherapies, spanning from the 1960-ies and 50 years onward, Weisz and colleagues [20] found that the interventions were usually delivered in settings outside regular clinical practice, i.e. in research settings. Across the trials, only 2.1% of all study groups were described as involving clinically referred clients treated by practitioners in regular clinical practice settings [20]. When delivered in regular clinical practice, evidence-based treatments (EBT), compared to treatment as usual (TAU), has modest outcome (ES, d = 0.29) [29]. Furthermore, in several instances TAU delivered in regular clinical practice, outperformed standard EBT, usually delivered as single-disorder interventions. Even studies using exclusively diagnosed samples (d = 0.09) and studies on clinically referred youths (d = 0.17) showed low and non-significant ES values [34]. Despite the importance of quality assurance in routine practice, most CAMHS do not evaluate patients clinical change systematically [35]. A report from the Child and Outcomes Research Consortium (CORC) 2013–2016 with patients receiving treatment over six months in one of our participating CAMHS, showed improvement in many patients, however as many as 27% deteriorated [36].

In a CAMHS setting, there are high production requirements for staff, so the treatments need to be short and effective. Clinicians in a managed care setting reportedly emphasize short-term cognitive behavioral strategies [37]. Transdiagnostic treatment focuses on treatment strategies that may be generic across diverse conditions and can be defined as a therapy made available to individuals with a wide range of disorders [38]. Transdiagnostic treatment is characterized by a focus on cognitive, behavioral, and physiological processes that are shared or common across diverse disorders [38]. Although focal EBT are excellent in many ways (see 20), there may be challenges associated with implementation of several disorder-specific CBTs in regular clinical practice, and hence reasons for advocating training in one transdiagnostic CBT intervention that spans over several disorders or symptom clusters. In the framework of regular clinical practice, transdiagnostic CBT could be more applicable, time-saving, realistic to learn and cost-efficient for therapists in terms of training and application, and last but not least, it addresses the comorbid states we encounter in regular practice (e.g. 20,33).

There is a growing body of evidence demonstrating that transdiagnostic treatments could be effective in the reduction of symptoms of anxiety and depression [39], furthermore that transdiagnostic CBT has similar effects as disorder-specific interventions [40], and finally that effect sizes range from medium to large for these types of interventions [41]. The Unified Protocol for the Treatment of Emotional Disorders in Adolescents (UP-A) showed a significant effect compared to waiting list controls on all outcome measures [26]. However, as highlighted in Weisz and colleagues [20] extensive meta- analysis, the vast majority of the 1160 treatment and control groups included therapy that was not delivered in regular clinical care settings.

To the best of our knowledge, the present study is the first RCT performed with short-term transdiagnostic CBT for adolescents, the SMART protocol, with combined emotional disorders in regular clinical settings in CAMHS.

### Objectives

The objective of the present study was to examine the effectiveness of a short-term, transdiagnostic CBT (SMART) in adolescents with clinically significant emotional symptoms referred to community clinics. The effectiveness is investigated both with regard to

- emotional problems as defined by the SDQ,
- symptoms of depression,
- symptoms of anxiety
- general functioning
- and general clinical status.

## Methods

The study is a randomized controlled study of the effects after 6 weeks of Structured Material for Therapy (SMART) treatment, compared with a waiting list control (WLC).

### Participants

**Fig. 1:**
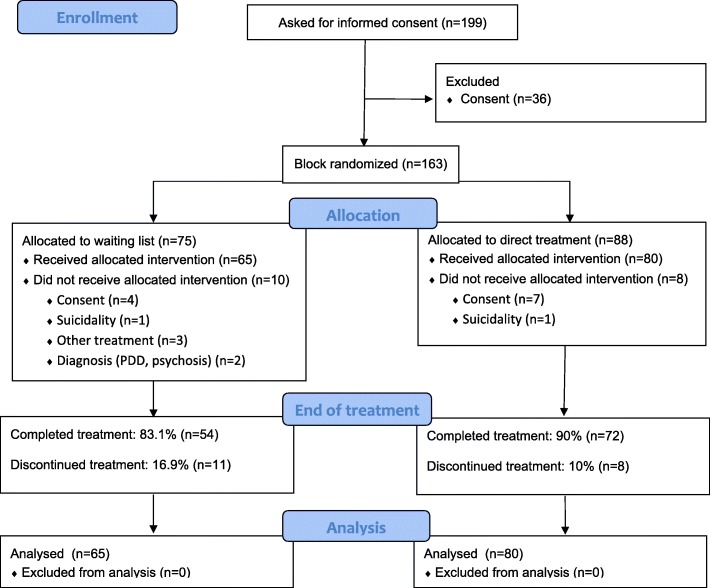
Consort flow diagram

The analyzed sample comprised of 145 adolescents 14–17 years old (*M* = 15.72, *SD =* 1.14, 90.3% females), recruited from referrals to three Norwegian public child and adolescent mental health outpatient clinics (CAMHS) between January 2012 and November 2016. Participants were informed about the study during the routine intake procedure of the clinic. All adolescents, parents of children under 16 years, and adolescents over 16 years signed informed consent and received the Strengths and Difficulties Questionnaire (SDQ). Inclusion criteria were [1] age between 14 and 17 years [2]; a probable diagnosis of emotional disorder as indicated by a score of at least 6 on SDQ emotional problems subscale; and [3] maintenance of a maximum waiting time for necessary medical care of 6 weeks given by Norwegian health authorities. Exclusion criteria were [1] a diagnosis of pervasive developmental disorder (PDD) [2]; psychotic symptoms [3]; Use of anxiolytic or anti-depressant medication effects during the treatment period; and [4] patients who did not speak the Norwegian language. A total of 199 adolescents were assessed for eligibility and were asked for informed consent. Of these, 36 did not consent, 7 were excluded due to exclusion criteria [1–4], 11 withdrew from the study. A total of *N* = 163 were block randomized into direct treatment, or six-week waiting list. In the current study, 19 patients did not complete the treatment. Of these, we had no information on the reason for non-completion for 11 patients, while 2 cited lack of motivation, 3 were referred to other treatment (2 received trauma treatment, 1 regular cognitive behavioral therapy), and finally 3 withdrew because of geographical distance (2 moved to another location, 1 had a long distance to travel to get to the CAMHS). (See CONSORT flow diagram in Fig. 1).

### Ethics, consent, permissions

The study was performed in compliance with the Helsinki Declaration for research on humans and was approved by the Regional Committees for Medical and Health Research Ethics (REC North, Reference number 2011/1937).

All participants participated and consented according to the regulations in the research project, also with written parental consent for those under age 16 (REC North, Reference number 2011/1937). Consent to publish was given from every participant and parents when warranted. The study adheres to CONSORT guidelines.

### Measures

#### Diagnostic instruments for study entry

***Development and Well Being Assessment (DAWBA***) [42], Norwegian language version [43], was a part of intake procedures for all patients at the participating clinics. DAWBA is a web-based diagnostic interview that is multi-informant with both open- and closed-ended questions. In this study, only information from the patients was used to assess diagnosis. When completed online, DAWBA uses computer algorithms to suggest the likelihood of diagnoses. DAWBA covers diagnoses in band levels corresponding to the prevalence of the disorder [44]. The bands range from levels 0–5 and are dichotomously combined to either ‘absent’ (levels 0–3; < 0.1 to 15% probability of disorder) or ‘present’ (levels 4–5; ~ 50 to > 70% probability of disorder). Goodman and colleagues [42] found that DAWBA could discriminate between community and clinic samples of youth. Goodman et al. [44] found that the DAWBA bands were well suited to find an approximate prevalence of disorders. Comparing the computer-generated DAWBA bands to clinician-rated diagnoses, Goodman et al. [44] found that DAWBA underestimates the actual prevalence on a group level. Agreement on an individual level showed kappa values that were usually between 0.4–0.7, sensitivity 0.4–0.8, specificity 0.98–0.99, positive predictive values 0.5–0.8 and negative predictive values 0.96–0.99.

***The Strengths and Difficulties Questionnaire (SDQ)*** [45], Norwegian language version [46]. To measure emotional problems the SDQ was completed as part of the DAWBA package at the times of enrollment and end of therapy. The version used was the self-rated SDQ for 11 to 17-year-olds with five subscales. The emotional problems subscale was the main inclusion criteria and the primary outcome measure for emotional symptoms. SDQ is an emotional and behavioral screening questionnaire, using a 3-point Likert scale, from 0 (not true) to 1 (somewhat true) to 2 (certainly true), giving a maximum score of 10 on the emotional symptom subscale. Goodman [45] suggested a cutoff on the emotional problem subscale of 6/7. SDQ is a frequently used screening instrument and has satisfactory psychometric properties [45–47]. In this study, we only used the emotional symptoms subscale, which has shown acceptable reliability and adequate internal consistency [46]. Internal consistency in our sample was acceptable (Cronbach’s *α* = .70).

***The Children’s Global Assessment Scale (CGAS)*** [48], Norwegian language version [49], was used as a secondary outcome measure for general level of function and was scored at enrollment and end of therapy. CGAS is a therapist-scored rating scale of global functioning ranging from 0 to 100, with higher scores indicating a higher level of function. To ensure the stability of the CGAS scores, each child’s clinical profile was scored blindly by a group of at least 3 trained clinicians, and the average score was employed. The clinicians had extensive experience with CGAS, having used it routinely in clinical practice. CGAS has shown good psychometric properties [50]. In the present sample, there was a high degree of reliability between CGAS raters (ICC = .97).

***Clinical Outcome in Routine Evaluation-Outcome Measure (CORE-OM)*** [51], Norwegian language version [52], was used as a secondary outcome measure for general symptom pressure and risk of suicide and self-harm and was distributed at enrollment and end of therapy. CORE-OM is a 34-item questionnaire with items using a 5-point Likert scale from 0 to 4, with higher scores indicating an increased symptom pressure.

Skre et al. [52] suggested a cutoff point of 1 for discriminating between clinical and non-clinical populations. CORE has a reader interface age of 14 years in Norwegian adolescents [52] and can thus be applied in this sample. CORE has shown good psychometric properties [52, 53]. A validation study on a Norwegian sample concluded that CORE-OM has the same psychometric properties as the English version [52, 53]. The internal consistency for the CORE-OM total score in the present sample was excellent (Cronbach’s *α* = .92).

***Beck Depression Inventory, second edition (BDI-II)*** [54], Norwegian language version (not yet validated on a Norwegian youth sample), was used as a secondary outcome measure for extent and depth of depression and were distributed at enrollment and end of therapy. BDI-II is a 21-item self-report questionnaire from ages 13 to 80 years. Items are rated on a 4-point Likert scale from 0 to 3, giving a maximum score of 63 [54]. suggest cutoff ranges between 14 and 19 for mild depression, 20–28 for moderate depression and 29–63 for severe depression. BDI-II has shown good psychometric properties [55–57]. The internal consistency for the BDI-II in the present sample was excellent (Cronbach’s *α* = .91).

***Multidimensional Anxiety Scale for Children (MASC)*** [58], Norwegian language version [59], was used as a secondary outcome measure for degree of anxiety and was distributed at enrollment and end of therapy. MASC is a 39-item self-report questionnaire for children and adolescents between ages 8 and 17 years. Items are grouped in 6 subscales and rated on a 4-point Likert scale from 0 to 3, with higher scores indicating a higher degree of anxiety. In the present study, we used the total score, converted to a t-distribution centered at approximately 50. MASC has shown good psychometric properties [58, 60]. The internal consistency of the MASC total score in the present sample was good (Cronbach’s *α* = .88).

***Evaluation of sessions*** [61] was employed as a measure of treatment integrity, alliance and user satisfaction and was distributed after each session and at the end of therapy. In the SMART manual, there is an evaluation after each session, where the patient rates aspects of each session on topics of specific content and satisfaction with the session on a Likert scale from 1 “very unsatisfied” to 5 “excellent”. The running aim is that the therapist adjusts therapy, alliance, content and relevance in collaboration with the patient.

### Procedure

Assessments were completed by youth pretreatment, post-waitlist and post-treatment. A block randomization was used in which groups of 5 youths meeting the inclusion criteria were randomized with a 1/1 chance to either waiting list control (WLC) or direct treatment. A random number generator in SPSS was used in the randomization procedure drawing numbers from the Bernoulli distribution. The participants were enrolled and assigned to treatment or WLC by administrative staff so that both the therapist, researchers and the participants were blinded to the allocation process. The mean duration of WLC was 6.8 weeks, and the mean duration of the treatment condition was 10.3 weeks. The most common explanations for prolonged treatment time were summer vacations, therapist sick leave and patients not showing up for treatment.

#### Treatment

The adolescents were treated with the SMART program [61], a Norwegian version of the GO! program, originally developed and evaluated in Germany [62]. SMART is developed for 14–25 years old adolescents and younger adults. SMART is an 8-week manual-based modularized CBT program, based on well supported methods for treating anxiety and depression with a strong emphasis on cognitive restructuring, exposure and activation. The special features of the program are as follows: definition of individual treatment goals, activation of personal resources, behavioral experiments, information about emotional problems and related coping strategies. The materials are organized in five modules (introduction, depression, anxiety, assertiveness training, and summary, in a total of eight sessions). The modularized organization of the materials allows for the program to be shortened to four or six sessions by selecting modules.

In this study, all modules except the assertiveness module (2 sessions) at the end of the program were employed as a standard brief therapy in the outpatient clinics. The reason for not employing the assertiveness module was that the WLC and the treatment group should initially be of the same duration, and we chose the modules that were targeting depression and anxiety symptoms. Four modules were delivered over 6 sessions, each with a duration of 1.5 h (see Table 1).

**Table 1:** Questions used as measures on treatment integrity

Question	Satsifaction ^a^	Adherence^b^
Introduction		
Introductory exercise	4.36 (0.19)	35.3%
Homework	4.07 (0.78)	90.4%
**Total**	**4.13 (0.74)**	**62.6%**
Module 1 (depression)		
Repetition/Homework	4.16 (0.73)	100%
Convolute exercise	3.35 (1.28)	65.4%
Cognitive distortion	4.12 (0.74)	97.8%
Looking for proof	4.15 (0.76)	94.9%
Information about depression	4.43 (0.60)	98.5%
Attributional error	4.17 (0.72)	95.6%
**Total**	**4.12 (0.77)**	**92.0%**
Module 2 (anxiety)		
Repetition/Homework	4.15 (0.70)	97.1%
Information about anxiety	4.47 (0.56)	100%
Anxiety circle	4.28 (0.64)	97.8%
Information about anxiety disorders	4.44 (0.58)	96.3%
Experiment with panic disorder	3.92 (1.00)	47.1%
Relaxation exercise	4.31 (0.88)	65.4%
**Total**	**4.31 (0.54)**	**83.9%**

*Note* a. Satisfaction ratings (*M* and *SD*), b. Adherence in %, 100 being full adherence

#### Treatment integrity

The therapists had a 2-day training course in the use of the SMART manual. The training consisted of lectures, hands-on training, and role play. When therapists started using the SMART manual, they had bi-weekly supervision based on the Cognitive Therapy Adherence and Competence Scale (CTACS) [63]. The supervision was mainly performed on Skype by the first and the last author who are trained and experienced CBT therapists and supervisors. CTACS is a widely used 21-item scale that measures therapist competence and adherence, and was here a part of the training and supervision of treatment integrity. In advance of each supervision session, the supervisor and therapists scored a video session with the CTACS. The session was then used to discuss the scores. To further strengthen the treatment integrity over time, periodic booster sessions where all therapists gathered for a full day meeting were arranged. The booster sessions were split between therapists presenting their way of working with the materials and sessions with supervision based on video records. As a measure of treatment integrity, scores from the *Evaluation of sessions* questionnaire where patients rated specific elements of each session were calculated (Likert scale from 1 “very unsatisfied” to 5 “excellent”). From this, an adherence score was computed where present versus missing ratings reflected the degree (in percentage) to which each element in the SMART manual was followed in the sessions (see Table 1).

#### Alliance and user satisfaction

The following items from the Evaluation of sessions questionnaire were used as a measure of therapeutic alliance: “I liked today’s session” and “I felt understood by the therapist” (Likert scale from 1 to 5, ranging from negative to positive). Three items from the end of the therapy questionnaire were used as measures of alliance: “Therapist’s competence and presentation were”, “Therapist’s understanding was” and “Therapist’s openness was” (Likert scale from 1 to 5, ranging from negative to positive).

As a measure of user satisfaction, two questions from the *Evaluation of therapy* questionnaire: “Overall the course was” (Likert scale from 1 to 6, ranging from negative to positive) and “I would recommend this course to others” (Yes/No).

#### Setting, therapists and assessors

The study was conducted in three public child and adolescent mental health outpatient clinics, covering both urban and rural parts of northern Norway. Adolescents are usually referred by general practitioners. Teams are multidisciplinary and work with a variety of disorders. Twenty therapists participated (*M* age = 39.18 years, *SD* = 10.93, range 24–57, 100% females). The therapists had 6.8 years of clinical experience on average (*SD* = 8.23, range 0–32 years). Of the 20 therapists, 11 were psychologists, 2 psychology students, 4 pedagogues, 2 social educators and 1 was a public health nurse. Two therapists had a two-year specific education and training in CBT.

### Data analysis

#### Power calculations

Initially, a necessary sample size of 160 patients was calculated based on two considerations: calculation of statistical power and expected attendance rate. The minimum required sample size for the comparison of group averages in two groups (two-tailed t-test with a 0.01 significance level, statistical power 0.80 and expected effect size on difference in mean scores between groups *d* = 0.60) was calculated to be 58–67 patients in each group [64]. The choice of the estimated effect size was based on the study by Weisz and colleagues [65], who in a summary of existing research on CBT with children and adolescents found an average effect size of 0.67. An expected attendance rate of 80% was based on data from a counselling service for young people in the same geographic area that had a no-show rate of 12–17% [66]. To treat the necessary 126 patients, we thus needed to recruit 160.

The results were reported as an intention-to-treat analysis [67] as suggested in the CONSORT 2010 statement [68]. Missing data for outcome variables were imputed using the multiple imputation (MI) procedure imputing 50 different datasets. MI has been suggested to be the recommended imputation technique when it is reasonable to assume that data are missing at random [69]. Imputations of missing data were based on predictive mean matching using the MICE package for R [70]. Each imputation was selected from a random draw among the 5 observations that were closest to the value predicted by the imputation model. Both demographic and outcome variables were used to predict (other) outcome variables. The linear mixed models procedure analyzed each of the 50 imputed datasets separately, and the results were pooled using standard procedures.

To test for the effects of the SMART treatment, linear mixed model analysis [71] was used. The data are hierarchical with measurement occasions (level 1; pre and post) nested within individuals (level 2). A random intercept was included in the model, but no random slope was included because of only two measurement occasions. A test of the significance of the time by group interaction is then a test of whether the SMART group and the control group change differently from pre- to post-treatment. A separate analysis was done which adjusted for the individual probability of being randomized directly into treatment (the SMART group). This analysis showed no difference from the main analysis.

Effect sizes with 95% confidence intervals were computed as a standardized difference between the group’s gain scores (Hedges’ g), using the pooled standard deviation of the pre-measurement for the standardization [72].

Pretreatment differences between the groups were tested using linear mixed models, one-way ANOVA, or chi-square tests, depending on the situation. The same methods were used to compare dropouts after pretreatment with non-dropouts on demographic variables and pretreatment outcome variables.

The Reliable Change Index (RCI) [73] was used to assess clinical and significant change on the SDQ.

We used IBM SPSS v24 for all analyses, and .05 was generally set as the significance level.

## Results

### Treatment integrity and user satisfaction

The adherence scores in Table 1 indicate a general high level of adherence to the manual, with a high completion percentage of the elements in the SMART manual. However, for four elements, the scores indicate that they were used to a lesser degree: “Introductory exercise”, “Convolute exercise”, “Experiment with panic disorder” and “Relaxation exercise”.

Table 2 shows demographics, diagnoses and comorbidity. The majority of patients were female, and there were too few boys in the sample to compare between genders.

**Table 2:** Demographics and diagnoses

	Total		WLC		Direct treatment	
Dawba prediction	*n*	% (of 145)	*n*	% (of 65)	*n*	% (of 80)
Only anxiety	34	23.4	18	27.7	16	20.0
Only depression	29	20.0	12	18.5	17	21.3
Depression and anxiety	46	31.7	18	27.7	28	35.0
Depression and GAD	30	20.7	10	15.4	20	25.0
Depression and Social phobia	27	18.6	10	15.4	17	21.3
Depression and specific phobia	7	4.8	0	0.0	7	8.8
Depression and agoraphobia	9	6.2	4	6.2	5	6.3
Depression and panic disorder	6	4.1	4	6.2	2	2.5
Neither anxiety nor depression	36	24.8	17	26.2	19	23.8
No diagnosis	9	6.2	5	7.7	4	5.0
Depression	78	53.8	24	36.9	54	67.5
Mild (ICD-10)	2	1.4	2	3.1	0	0.0
Moderate (ICD-10)	27	18.6	10	15.4	17	21.3
Severe (ICD-10)	39	26.9	12	18.5	27	33.8
Unknown	10	6.9	0	0.0	10	12.5
Generalized anxiety disorder	36	24.8	14	21.5	22	27.5
Social phobia	45	31.0	20	30.8	25	31.3
Specific phobia	24	16.6	7	10.8	17	21.3
Agoraphobia	14	9.7	7	10.8	7	8.8
OCD	4	2.8	3	4.6	1	1.3
Panic disorder	9	6.2	6	9.2	3	3.8
Total n	145	100	65	100.0	80	100.0

*Notes.*

*Diagnoses in both ICD-10 and DSM-IV (same algorithm or same number of diagnoses)*

A quarter of participants had a probable pure anxiety disorder, one fifth had a probable pure depressive disorder and one third had a probable diagnosis of both anxiety and depression. One fifth had other disorders, and 10 % did not reach the probability level of any diagnosis. Severe depression (ICD-10) was diagnosed in more than half the participants with a diagnosis of depression.

### Alliance

The scores on questions of alliance indicate that the patients liked the sessions, felt understood by the therapist both after sessions and at the end of therapy (see Table 3).

**Table 3:** Questions used as measures on alliance

Question	*n*	Average score: M (SD)
End of session questionnaire		
I liked today’s session	117	4.60 (0.60)
I felt understood by the therapist	120	4.43 (0.69)
End of therapy questionnaire		
Therapist understanding was	119	4.61 (0.69)
Therapist openness was	120	4.60 (0.76)

*Note.* Scores on a Likert scale from 1 to 5, where 5 is most satisfied

Scores based on means across all sessions

### Pre-treatment differences between conditions and change from pre to post therapy

Group differences between the two treatment conditions were compared at baseline on the outcome variables. Differences between the groups on all variables at baseline were non-significant (see Table 4).

**Table 4:** Summary results for main outcome variables in each treatment condition

	SMART		Wait list		Group effect^a^	Effect size
	*n*	mean (sd)	*n*	mean (sd)		g (95% CI)
SDQ emotion (youth)						
Pre	80	7.89 (1.45)	65	7.99 (1.19)	t = −0.30, n.s.	
Post	80	6.22 (2.65)	65	7.18 (2.00)	t = 2.06, p = .039	0.65 (0.31, 0.98)
CGAS						
Pre	80	52.08 (8.97)	65	49.58 (7.69)	t = 1.35, n.s.	
Post	80	60.68 (12.33)	65	53.48 (11.81)	t = 2.35, p = .019	0.56 (0.23, 0.89)
MASC total						
Pre	80	61.10 (11.57)	65	62.72 (9.76)	t = −0.84, n.s.	
Post	80	54.09 (13.26)	65	59.42 (10.99)	t = 2.10, p = .035	0.34 (0.01, 0.67)
BDI						
Pre	80	28.98 (12.48)	65	29.19 (10.91)	t = −0.10, n.s.	
Post	80	20.52 (14.37)	65	24.23 (10.84)	t = −1.84, p = .066	0.30 (−0.03, 0.63)
CORE total						
Pre	80	1.93 (0.65)	65	2.04 (0.47)	t = −1.07, n.s.	
Post	80	1.49 (0.68)	65	1.71 (0.62)	t = −1.06, *p* = .289	0.19 (−0.13, 0.52)

*Note* a. At pretreatment, main effect of group (SMART/Wait-list); At post-treatment, group interaction effect (time * group)

#### Self-reported emotional problems (SDQ emotional problems subscale)

There was a significant time by group interaction on the SDQ emotional scale (see Table 4). While the treatment group had a mean decrease of 1.67 points, the wait-list group had a smaller change (0.81 points), and the effect can be classified as medium (*g* = 0.65 *p =* .039).

#### General functioning (CGAS)

The two conditions showed significantly different pre-post changes on the CGAS (see Table 4). While the treatment group had a mean increase of 8.6 points, the wait-list group had a smaller change (3.9 points), and the effect can be classified as medium (*g* = 0.56, *p =* .019).

#### Anxiety (MASC)

For the MASC total score, there was a significant time by group interaction, and the treatment condition had a better development (decrease of 7 points) than the wait-list condition (decrease of 3.3 points), and the effect can be classified as small (*g* = 0.34, *p =* .035) (see Table 4).

#### Depression (BDI-II)

There was no significant group difference on the BDI-II total score between the treatment (decrease of 8.46 points) and the wait-list condition (decrease of 4.96 points), *g =* 0.30, *p* = .066 (see Table 4).

#### General symptom pressure and risk of suicide and self-harm (CORE-OM)

There was no significant group difference on the CORE-OM total score between the treatment (decrease of 0.44 points) and the wait-list condition (decrease of 0.33 points), *g* = 0.19, *p* = .29.

#### Reliable change index and clinically significant change

Of 62 patients eligible for this analysis in the treatment group (SMART), 17.7% (*n* = 11) experienced clinical and reliable change (see Table 5). Of 52 patients eligible for this analysis in the waiting-list condition, 5.8% (*n* = 3) experienced clinical and reliable change. Furthermore, 16.1% (*n* = 10) of the patients in the treatment condition experienced either clinical, or reliable change, as compared to 5.8% (n = 3) of the waiting-list patients. No patients in either group showed deterioration.

**Table 5:** Summary of reliable change and clinical change

	SMART^a, b, c^		Wait list	
	*n*	%	*n*	%
Clinical and reliable change	11	17.7%	3	5.8%
Only clinical change	9	14.5%	3	5.8%
Only reliable change	1	1.6%	0	0%
No change	41	66.1%	46	88.5%
Deterioration	0	0%	0	0%
Total	62		52	

a. *Group difference on clinical change* vs. *non-clinical change: Fisher’s exact test p = 0.013. b. Group difference on no change* vs. *clinical change or reliable change: Fisher’s exact test p = 0.007. c. Group difference on clinical and reliable change* vs. *not both clinical and reliable change: Fisher’s exact test p = 0.084*

*Note*

Reliable change = 4 point improvement on the SDQ emotional scale

Clinical change = from above to below cutoff

Clinical and reliable change = both

Deterioration = 4 point increase on the SDQ emotional scale

## Discussion

To the best of our knowledge, this is the first RCT testing the effect of a 6-session transdiagnostic CBT treatment in an adolescent sample suffering mainly from combined emotional disorders, receiving the intervention in a CAMHS naturalistic setting. Despite that this brief manualized treatment was delivered over only six sessions, the treatment condition showed statistically significant treatment effects for internalization symptoms, anxiety symptoms and general functioning compared to waiting-list controls. Clinically significant change in emotional problems (SDQ) was observed significantly more frequently in the treatment condition.

The effect sizes of the intervention on the measures of emotional problems, general functioning and anxiety symptoms were moderate to small. Furthermore, both depressive symptoms and general clinical outcome changed in the desired direction, albeit showing no statistically significant differences between the treatment and wait-list conditions. Finally, the users report of satisfaction and alliance indicate that the treatment was well received for the adolescents.

The recovery rates in this study were lower than what is expected for CBT efficacy trials for single-disorder treatments [20] where the majority of studies show moderate to large effects targeting anxiety and depression [21, 22]. However, with SDQ emotion (g = 0.65) and MASC (g = 0.34) for anxiety this trial shows comparable effects to [20] and lays slightly above the effects shown by Weisz and colleagues [34] in real-world settings. Compared to effectiveness studies and the effect sizes in the context of studies performed in ordinary clinical practice [34], the present effect sizes are promising, keeping in mind the brief duration of the intervention. Considering the rate of change on the SDQ and CGAS, compared to the total of patients receiving treatment in the participating CAMHS after six month of treatment [36], our sample shows comparable rates of change after six session of treatment, 10 weeks with a decrease of 2.6 points on the SDQ total score and an increase of 9.6 points on the CGAS.

Using the RCI [73] as a measure of clinical significant change, nearly one-sixth of the youths receiving treatment in the present study obtained clinical meaningful change, none deteriorated, while nearly two-thirds showed no reliable change. Although the proportion who experienced partial or full recovery may seem modest, there are indications that the majority of children and adolescents in regular community mental health care do not experience clinical improvement applying these conservative criteria [74, 75]. In addition, the adolescents in the treatment condition improved nearly one category on the CGAS indicating clinically meaningful change after a brief intervention. Some studies from ordinary care report poorer outcome following treatment [76, 77]. In the CORC report mentioned earlier 27% of the CAMHS patients showed deterioration [36]. No patients in either group in this study showed worsening.

The treatment program and the inclusion of both therapists and patients have shown good feasibility and transportability to ordinary clinical practice. Firstly, the therapists in this study were representative of clinical practice with their diverse educations and years of occupational experience, where most of them had limited experience with CBT beforehand. Secondly, the treatment is of short duration and adherence to the manual components was satisfying. In addition, the patients rated the alliance and their satisfaction with the program as good.

Keeping these characteristics in mind, the SMART program could be considered as a first step in a clinical stepped care delivery followed by more intensive evidence-based treatments for single disorders, e.g., the C.A.T. program [78] for anxiety and more intensive programs for depression.

### Strengths and limitations

The strengths of the present study were that it was performed in an ordinary clinical setting, by therapist’s representative for regular CAMHS practice, with regular referred patients. Furthermore, the sample size is fairly large, and the study has good statistical power to detect moderate to large effects. The data quality is good, with a nearly complete data-set from pre- to post-therapy and measures for treatment integrity. The study included a number of outcome measures, including adherence and acceptability.

A possible limitation of the study was that the mean duration for treatment condition was 3.5 weeks longer than for the WLC, which could be in favor of the intervention. Another possible limitation regarding representativeness is that the governmental restrictions on waiting time prevented us to enroll a part of referred patients in the study. However, in the mentioned report from the Child and Outcomes Research Consortium (CORC) concerning the total of patients receiving treatment in one of our participating CAMHS, from 2013 to 2016 [36], the SDQ total score and the CGAS score before treatment was lower in the total population of CAMHS than in our sample [36].These scores indicate that more acute and serious disorders was not underrepresented in our sample. With this in mind, the results should be interpreted with caution. Regarding representativity, there were too few boys (*n* = 14) to perform analysis of gender differences. Recent studies show evidence of partial gender non-equivalence with a tendency for girls to more often endorse items measuring symptoms of emotional problems and prosocial behavior on the SDQ [46]. This could have implications for the SDQ as a screening instrument and could have affected the recruitment of boys in the study, indicating a lower cut-off for inclusion of boys. Another limitation is the use of a waiting list as a control condition as opposed to an active control condition. However, the intention was that the two experimental conditions have equivalent length. To create equal length between the conditions the assertiveness module in SMART was removed. This was warranted to accommodate the ethical and legal issues concerning that the patients could not wait more than 6 weeks, however this conflates time and treatment and limits the ability to ascertain the comparative effect of full-scale SMART treatment. SMART is both transdiagnostic and modularized, however in this study SMART was delivered in a linear order to all patients. Some results show that delivering in a modular, flexible fashion gives better results [34]. Although SMART shows promising results, the effectiveness should be qualified as preliminary, requiring future evaluation of the full-scale program in a modularized format to assess “active ingredients” as well as predictors of treatment response and assessment of long-term effects. The treatment should also be compared to an active control group.

## Conclusions

Results from this RCT are promising and indicate support for the effectiveness of a transdiagnostic short-term CBT compared to no intervention for youth with emotional problems in community clinics with only 6 sessions of treatment. The recovery rates highlight the need for further improvement for some of the patients. Considering that the treatment is very short, only two sessions of CBT for depression and two for anxiety transdiagnostic treatment, SMART can with further investigations be considered as first step in a stepped care model of treatment of anxiety and/or depression in CAMHS. Further evaluation is needed of the full-scale program and to find the optimal combination with additional interventions.

## Data Availability

The datasets generated and analysed during the current study and the full study protocol are available from the corresponding author on reasonable request.

## Abbreviations

BDI II: Beck Depression Inventory, second edition
CAMHS: Child and Adolescent Mental Health Services
CBT: Cognitive Behavior Therapy
CGAS: The Children’s Global Assessment Scale
CORC: Child and Outcomes Research Consortium
CORE-OM: Clinical Outcome in Routine Evaluation-Outcome Measure
DAWBA: Development and Well Being Assessment
EBT: Evidence Based Treatment
ES: Effect Sizes
IPT: Interpersonal Therapy
MASC: Multidimensional Anxiety Scale for Children
PDD: Pervasive Developmental Disorder
RCI: Reliable Change Index
RCT: Randomized Controlled Trial
SDQ: Strength and Difficulties Questionnaire
SMART: Structured Material for Therapy
TAU: Treatment as Usual
UP-A: The Unified Protocol for the Treatment of Emotional Disorders in Adolescents
WLC: Waiting List Control
